# Enhanced surveillance for tuberculosis among foreign-born persons, Finland, 2014–2016

**DOI:** 10.1186/s12889-018-5501-y

**Published:** 2018-05-09

**Authors:** Pirre E. Räisänen, Hanna Soini, Pirjo Turtiainen, Tuula Vasankari, Petri Ruutu, J. Pekka Nuorti, Outi Lyytikäinen

**Affiliations:** 1 National Institute for Health and Welfare (THL), Department of Health Security, Helsinki, Finland; 2 Department of Epidemiology, Health Sciences, Faculty of Social Sciences, University of Tampere, Tampere, Finland; 3 Finnish Lung Health Association (Filha), Helsinki, Finland; 4 Department of Pulmonary Diseases and Clinical Allergology, University of Turku, Turku, Finland

**Keywords:** Tuberculosis, Screening, Immigrants, Asylum seekers, Refugees

## Abstract

**Background:**

Tuberculosis (TB) in foreign-born residents is increasing in many European countries including Finland. We conducted enhanced TB surveillance to collect supplementary information on TB cases among recent immigrants and their children to provide data for revising TB control policies in Finland to take into account the decrease in native cases and increase in foreign-born cases.

**Methods:**

TB cases were identified from the National Infectious Diseases Register. Data on foreign-born (if not available, most recent nationality other than Finnish) TB cases notified during 2014–2016 (country of birth, date of arrival to Finland, participation in TB screening, date of first symptoms, and details of possible contact tracing) were requested from physicians responsible for regional communicable disease control through a web-based questionnaire.

**Results:**

Questionnaires were returned for 203 (65%) of 314 foreign-born TB cases; 36 (18%) were paediatric cases TB was detected in arrival screening in 42 (21%) and during contact tracing of another TB case in 18 (9%); 143 (70%) cases sought care for symptoms or were identified by chance (e.g. chest x-ray because of an accident). Of cases with data available, 48 (24%) cases were diagnosed within 3 months of arrival to Finland, 55 (27%) cases between 3 months and 2 years from arrival, and 84 (42%) cases after 2 years from arrival. Of all the foreign-born cases, 17% had been in a reception centre in Finland and 15% had been in a refugee camp abroad.

**Conclusions:**

In addition to asylum seekers and refugees, TB screening should be considered for immigrants arriving from high TB incidence countries, since the majority of TB cases were detected among persons who immigrated to Finland due to other reasons, presumably work or study. Further evaluation of the target group and timing of TB screening is warranted to update national screening guidance.

## Background

Screening of immigrants arriving from high tuberculosis (TB) incidence countries (incidence 50/100000 or more) and timely detection of TB are key actions for preventing transmission and spread of pulmonary TB. Increasing international migration is thought to be the most important factor contributing to the increase in cases and trends observed in the epidemiology of TB in high income countries in Europe [1–3]. Studies conducted since the 1990s attribute a large proportion of cases in high income countries to foreign-born residents [3–6]. Only few studies have been published concerning TB among second generation immigrants in Europe [6–9].

In Finland, the proportion of new foreign-born TB cases increased from 6% in 1995 to 46% in 2016, suggesting that the epidemiology of TB is changing in Finland. The absolute number of cases also increased from 38 in 1995 to 106 in 2016 [10]. However, the number of immigrants living in Finland also increased from approximately 106,000 in 1995 (2% of the population, TB incidence 36/100000) to 360,000 in 2016 (7% of the population, TB incidence 29/100000). Countries with high TB incidence such as Somalia, Afghanistan, and Vietnam were the most common birth places of foreign-born persons living in Finland in 2016 [11].

According to national guidelines, refugees and asylum seekers are screened for TB within two weeks of arrival to Finland. TB screening is offered for persons arriving from high TB incidence countries (≥50/100000), conflict areas or refugee camps or, if they have TB symptoms. Screening includes an interview and a chest x-ray. Offering screening is mandatory but participation is voluntary. TB screening is also offered to other immigrants who come from countries with a very high TB incidence (≥150/100000), and who plan to stay in Finland for 3 months or longer. Routine screening for latent tuberculosis infection (LTBI) among immigrants is not recommended in Finland. Moreover, all children born to parents from high-incidence countries receive Bacillus Calmette-Guérin (BCG) vaccine at birth.

The TB screening policy for refugees and asylum seekers has been in effect since 2009. When large numbers of asylum seekers arrived to Finland in 2015–2016, the policy was changed to include also individuals arriving from conflict areas (such as Iraq and Syria) or refugee camps. The policy concerning other immigrants has been in effect since 2014 and was modified in 2016 (TB incidence threshold was increased to 150/100000 to increase cost-effectiveness).

Second generation immigrant TB cases have not been studied earlier in Finland. In other low TB incidence countries such as Sweden, Norway, Denmark and The Netherlands [5], TB cases in foreign-born dominate the epidemiology of TB. In these countries, the proportion of immigrant cases varies between 50 and 85% of all cases. As surveillance and control of TB needs to be revised and improved in Finland to take into account the decrease in native cases and increase in foreign-born cases, enhanced TB surveillance was launched to collect detailed information of TB among migrants and their children. Our aim was to find out: 1) how TB was detected, 2) when TB was detected relative to arrival to Finland, 3) were any TB symptoms reported, and 4) what was the proportion of TB cases who were asylum seekers and refugees.

## Methods

TB cases diagnosed from 1.1.2014 to 31.12.2016 were identified from the National Infectious Diseases Register (NIDR), a population-based surveillance system. All physicians and laboratories notify TB cases to the NIDR using ECDC criteria, notifying is mandatory [10]. The case definition for TB surveillance included all cases confirmed by culture, sputum smear, nucleic acid amplification or histology and also reporting category ‘physician’s decision to initiate full TB treatment on the basis of clinical suspicion of TB despite lack of laboratory confirmation’. Data collected from NIDR with each notification include age, gender, country of birth, nationality, date of diagnosis, and clinical presentation of TB (pulmonary/extrapulmonary). Categorical variables were compared with the χ2 test or Fisher’s exact test by Statistical Analysis System Software, version 9 (SAS Institute, Inc., Cary, North Carolina) [12].

A web-based questionnaire was sent to physicians responsible for communicable disease control in regions, where foreign-born or paediatric TB cases were notified. Data was requested on country of birth, date of arrival to Finland, travel route to Finland, whether the person has lived in a refugee camp before arriving in Finland, whether the person has stayed in a reception centre in Finland, participation in TB screening, date of first symptoms (e.g. cough, night sweats, weight loss, swelling or a lump), and possible contact tracing results. For paediatric cases, the parents’ country of birth was reported.

A foreign-born person was defined as a person born outside of Finland or, if the country of birth was not known, the most recent nationality was not Finnish. A paediatric case in this study was a person under 18 years old. A second-generation immigrant was defined as a person under 18 years old, born in Finland and having at least one foreign-born parent.

## Results

In total, 771 TB cases were diagnosed in Finland during the study period; 314 (41%) were foreign-born and 48 (6%) were paediatric cases (foreign-born or born in Finland).

### Foreign-born cases

The questionnaires were returned for 203 (65%) of 314 foreign-born TB cases notified in the NIDR during 2014–2016. The most common countries of birth were Somalia, Afghanistan and Vietnam (Table 1). The 203 patients were born in 43 different countries, and in one case the country of birth was unknown. Pulmonary TB was diagnosed in 140 cases (69%), and 9 of them (6%) had multidrug-resistant TB (MDR-TB). TB was detected in screening performed on arrival in 42 (21%) cases, 18 (9%) cases were found at contact tracing of another TB patient, and 143 (70%) cases sought care due to symptoms or were found by chance (e.g. chest x-ray taken due to an accident). The period from arrival to Finland to the date of diagnosis of TB was less than three months in 48 (24%) cases, three months to two years in 55 (27%) cases, and more than two years in 84 (42%) cases. For 16 (8%) cases, the time of disease onset was not available. Of all the cases, 35 (17%) had stayed in a reception centre in Finland, and 30 (15%) had been in a refugee camp prior to arrival in Finland.

**Table 1:** Characteristics of foreign-born TB cases, 2014–2016, Finland

	Foreign-born case (*n* = 203)^a^
Median age, years (range)	28.5 (2–83)
Male cases *n* (%)	120 (59)
Country of Birth	
Somalia *n* (%)	55 (27)
Afghanistan *n* (%)	15 (7)
Vietnam *n* (%)	14 (7)
Thailand *n* (%)	10 (5)
The Philippines *n* (%)	10 (5)
Other/unknown *n* (%)	99 (49)
Stayed in a refugee camp abroad *n* (%)	30 (15)
Stayed in a reception centre in Finland *n* (%)	35 (17)
TB found in screening *n* (%)	42 (21)
TB diagnosed within two years after arrival to Finland *n* (%)	103 (51)
Symptoms of TB *n* (%)	153 (75)
Pulmonary TB *n* (%)	140 (69)

^a^questionnaire data available

Of the 203 foreign-born TB cases 50 (25%) reported no symptoms compatible with TB. Thirteen (26%) of the asymptomatic cases were detected in screening performed at or soon after arrival, 10 (20%) due to being investigated because of an accident or death, and 12 (24%) during contact tracing. In 15 cases this information was not available. Of the foreign-born TB cases without symptoms, 42 (84%) had pulmonary TB, 9 (18%) had been living in a refugee camp on the way to Finland and 14 (28%) in a reception centre in Finland. The proportion of pulmonary TB was higher among asymptomatic cases (84%) than among symptomatic cases (54%). Forty-seven (94%) of the cases with no symptoms were born in a high TB incidence country.

### Paediatric cases

**Fig. 1:**
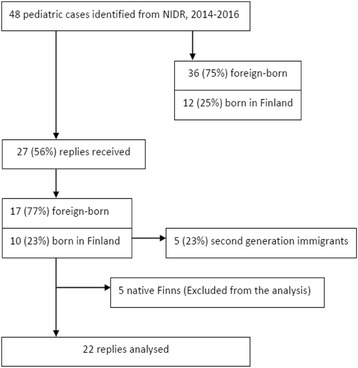
Flow chart of paediatric cases, 2014–2016

Between 2014 and 2016, 48 paediatric TB cases were identified from the NIDR. Of them, 36 (75%) were foreign-born and 12 (25%) were born in Finland. We received 27 (56%) replies for the questionnaire, and in 5 cases both parents were born in Finland. These cases were excluded from the analysis (Fig. 1).

Of the remaining 22 paediatric TB cases with questionnaire data available and at least one parent born outside Finland, 17 (77%) were foreign-born and 5 (23%) were born in Finland, classified as second- generation immigrants. Their parents were born in high TB incidence countries, except two of the fathers who were born in Finland. Six (27%) children were born in Afghanistan, 3 (14%) in Somalia and one (5%) in Bahrain, Ethiopia, Malawi, Myanmar, the Philippines, South Africa, Thailand and Vietnam, each.

Pulmonary TB was diagnosed in 19 children (86%), and 2 (11%) had MDR-TB. All Finnish-born paediatric cases (5) were identified during contact tracing of another TB patient and all had pulmonary TB. Of the foreign-born paediatric TB cases, 7 (41%) were identified in screening performed on arrival, 4 (24%) cases were found during contact tracing of another TB patient and 6 (35%) cases sought care due to symptoms or were found by chance (e.g. chest x-ray taken for another reason). The period from arrival to Finland to the date of diagnosis of TB, was less than three months in 9 (53%) cases, three months to two years in 4 (24%) cases, and more than two years in 4 (24%) cases. Eight children (47%) had resided at a reception centre in Finland, and 5 (29%) had been in a refugee camp prior to arrival in Finland.

No TB symptoms were reported for 22 paediatric TB patients 13 (59%). Five (38%) of them were found in screening performed at or soon after arrival, 7 (54%) in contact tracing and 1 (8%) was found by chest x-ray taken for another reason. All had pulmonary TB, one (8%) had MDR-TB, 4 (31%) had been living in a refugee camp abroad, 3 (23%) in a reception centre in Finland, and 10 (77%) were born in a high TB incidence country.

Seeking care due to symptoms was more common among foreign-born adults than children (*p* < 0.01) (Table 2). Early detection of TB (less than three months after arrival), asymptomatic disease, and staying at a reception centre were more common among children than adults (*p* < 0.01).

**Table 2:** Characteristics of adult and paediatric foreign-born TB cases, 2014–2016, Finland

	Adult *n* (%) (*n* = 186)^a^	Paediatric *n* (%) (*n* = 17)^a^	*p*-value
Pulmonary TB	125 (67)	14 (82)	0.198
Multidrug-resistant TB	7 (6)	2 (14)	0.099
Method TB detected			
Screening performed at arrival	35 (19)	7 (41)	0.029
During contact tracing of another TB patient	14 (8)	4 (24)	0.026
Sought care for symptoms or by chance	137 (74)	6 (35)	<0.01
Time from arrival to Finland to diagnosis of TB			
< 3 months	39 (21)	9 (53)	<0.01
3–23 months	51 (27)	4 (24)	0.73
≥ 24 months	80 (43)	4 (24)	0.119
Unknown	16 (9)	0 (0)	0.254
Person had resided in			
Reception centre in Finland	27 (15)	8 (47)	<0.01
Refugee camp abroad	25 (13)	5 (29)	0.076
No TB-related symptoms	40 (22)	10 (59)	<0.01

^a^questionnaire data available

## Discussion

This study shows that in three quarters of cases TB was detected when a foreign-born person was seeking medical care because of symptoms. Half of the cases were diagnosed within two years of arrival to Finland. Only one fifth of the TB cases were detected among individuals who had stayed in a reception centre in Finland and were classified as refugees or asylum seekers. Most of the detected TB cases (80%) were therefore among individuals who had come to Finland for other reasons, such as work or study.

Our study shows that 25% of the foreign-born cases had not reported any symptoms but yet TB was found in a chest x-ray taken for screening or contact tracing purposes. Thus, TB was detected before any symptoms occurred, and further transmission of the disease was avoided. Surprisingly, the proportion of pulmonary TB was higher among asymptomatic cases than symptomatic cases. This might be due to many reasons. First, extrapulmonary TB may cause pain and/or abscess in the affected part of the body and for that reason, person is seeking medical care. Second, pulmonary symptoms are not always recognized as TB, since some of the TB cases are first treated as pneumonia or common cold. In addition, early stages of pulmonary TB do not always cause noticeable symptoms especially among previously healthy young individuals. Third, the foreign-born patients may be reluctant to report their TB symptoms due to fear of stigma or deportation. Large proportions (60%) of the paediatric patients were reportedly asymptomatic. TB in children often presents with atypical symptoms and it is difficult for small children to communicate their symptoms. Since children primarily stayed at reception centres where screening practices were routinely implemented, their TB was detected earlier than adults.

Approximately 20% of TB cases in this study were found by screening performed at arrival to Finland. This suggests that immigrants coming from high TB incidence countries might benefit from chest x-ray screening even if they do not have any symptoms. Chest x-ray may be used to assess asymptomatic active disease [13]. In Finland, chest x-ray is combined with an extensive interview and symptom screening of the individual. Immigrant screening policies in other low TB incidence countries vary widely and may influence observed rates of TB [14]. Some countries i.e. Australia, Canada, New Zealand, the United States and the United Kingdom have a pre-entry screening policy [5, 15]. The United Kingdom and the Netherlands also have a post-arrival latent tuberculosis screening policy with an active tuberculosis screening policy [5].

In Finland, screening of TB among refugees and asylum seekers is routinely performed at reception centres and screening coverage is good [16]. Screening of other immigrants (such as students, workers, family members) arriving from high-TB incidence countries is also recommended, but not consistently implemented [17]. Since systematic screening of all immigrants is unrealistic, public-health efforts should be aimed at situations were transmission of TB is more likely to occur or would cause more serious consequences to the affected (such as schools, day-care centres and hospitals). The Finnish TB screening policy has recently been modified to target all health-care workers and caretakers of small children arriving from very high TB-incidence countries [18].

More than half of the foreign-born cases were diagnosed within two years after arrival to Finland. On the other hand, some cases had been living in Finland for ten years or more. We do not know whether these persons had travelled to their home countries or whether transmission, or reactivation of latent infection, occurred in Finland. Our previous studies using molecular genotyping methods show that TB was more often caused by reactivation of LTBI obtained in the country of origin of the patient, and that transmission of TB between Finns and foreign-born persons was rare [10, 19]. Moreover, we do not know if the diagnostic delay was due to delays in the health-care system or the patient’s care seeking. It is possible that the patient had sought help earlier but the possibility of TB was not investigated. Furthermore, persons from high TB incidence countries have a lifelong increased risk of TB. A similar scenario has been reported from Sweden where 23% of all immigrant TB cases had been living in the country for more than ten years [20].

In Finland, three quarters of children diagnosed with TB were foreign-born, 5 were second-generation immigrants and 5 cases were detected among children of Finnish origin. As the population of second-generation immigrants is much smaller, we may assume that the incidence rate among second-generation children is likely much higher than among Finnish children. [6].

A major limitation for our study was the low response rate. The questionnaire was sent to the physician in charge of communicable diseases, not directly to the clinician treating the patient. The patient data may not have been consistently recorded or requested data may have been missing. This is especially common for asylum seekers, who often move within one country and between countries, and their health records do not always follow. Moreover, asylum seekers do not receive the Finnish national identifier, which is needed for linking health records.

## Conclusion

Half of the reported TB cases among foreign-born persons were detected within two years of arrival; most of them were seeking care for TB-related symptoms. As less than 20% had been seeking asylum in Finland and therefore screened, most of the cases had arrived in Finland for other reasons such as work or study. Further evaluation of the target groups and timing of TB screening is warranted to update national screening guidance.

## Abbreviations

BCG: Bacillus Calmette-Guérin
Filha: Finnish lung health association
MDR-TB: Multidrug-resistant TB
NIDR: National infectious diseases register
TB: Tuberculosis
THL: National Institute for Health and Welfare
